# Supplementary material for: Exploring variability in the diet of depredating sperm whales in the Gulf of Alaska through stable isotope analysis
Source: R Soc Open Sci. 2020 Mar 11;7(3):191110. doi: 10.1098/rsos.191110 (PMC7137980; doi:10.1098/rsos.191110)
Supplement: Appendix 1 [file rsos191110supp1.docx]

**Appendix 1**

*Including ragfish in stable isotope analysis*

We attempted to collect ragfish in our experiment due to their prevalence in diets of sperm whales during commercial whaling, but were only able to obtain three samples, all of which were collected outside the study area. When we included ragfish in the isotope analysis, they fell in similar isotopic space as both sablefish and spiny dogfish (A1). Mixing models would have a difficult time isolating the overlapping prey sources, suggesting the ragfish be combined with sablefish and dogfish prey for analysis.


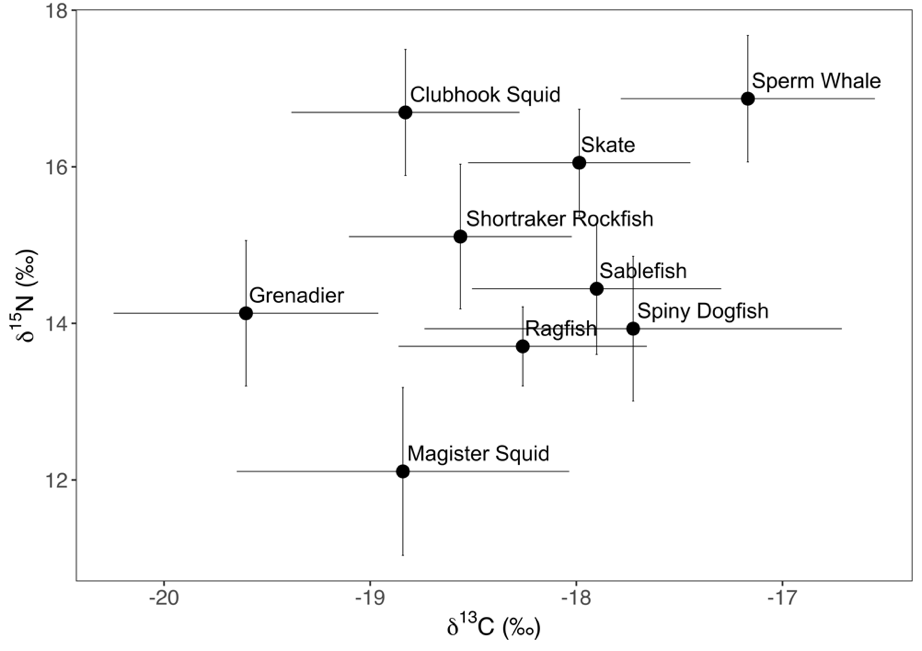


A1. Stable isotope ratios of sperm whales and presumed prey items, including ragfish, in the Gulf of Alaska. Points are means for each species, while error bars represent one standard deviation from the mean.

*Mixing model results including ragfish in models*

When mixing models were run with ragfish included, proportions of prey to sperm whale diets remained similar to results without ragfish (A2, A3). The sablefish/dogfish group, with ragfish added, remained the highest proportion of sperm whale diets, as well as skates (A2, A3).


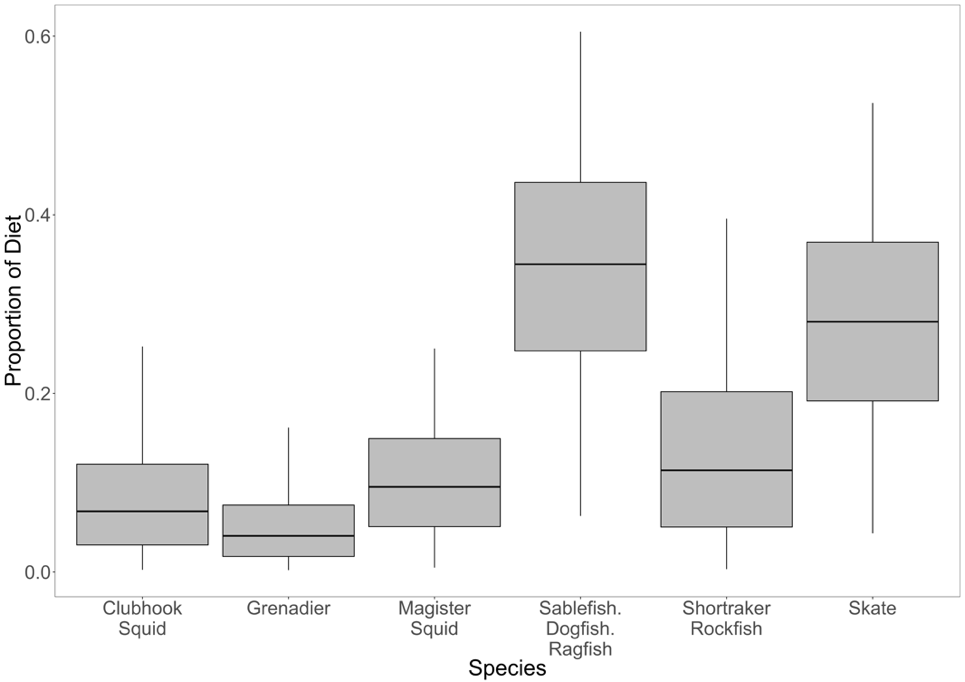


A2. Boxplots showing mixing model estimates of the proportional contribution of each prey to sperm whale diets, with ragfish included. Boxes represent lower and upper quartiles with a median line, while ends of whiskers show 95% credible intervals.

A3. Summary of estimated contributions (mean ± sd) of each prey item to sperm whale diets. Columns show all prey with sablefish & dogfish combined as seen in the main manuscript, all prey with ragfish added, all prey with sablefish and dogfish separated, all prey with a low end trophic enrichment factor (TEF) applied (1), and all prey with a high end TEF applied (2).

| **Species** | **Sablefish & Dogfish combined** | **Ragfish added** | **Separated Sablefish & Dogfish** | **Low TEF** | **High TEF** |
| --- | --- | --- | --- | --- | --- |
|  |  |  |  |  |  |
| Clubhook Squid | 8.3 ± 6.6 | 8.4 ± 6.7 | 9.4 ± 7.1 | 15.7 ± 10.0 | 7.3 ± 5.9 |
| Grenadier | 5.7 ± 4.7 | 5.2 ± 4.4 | 6.1 ± 4.9 | 6.9 ± 5.3 | 10.2 ± 7.5 |
| Magister Squid | 10.6 ± 6.8 | 10.4 ± 6.7 | 8.1 ± 5.7 | 7.3 ± 5.1 | 29.6 ± 8.7 |
| Sablefish.Dogfish.Ragfish | - | 34.2 ± 13.8 | - | - | - |
| Sablefish.Dogfish | 35.6 ± 13.9 | - | - | 17.8 ± 10.3 | 25.1 ± 12.7 |
| Sablefish | - | - | 22.7 ± 13.9 | - | - |
| Dogfish | - | - | 20.5 ± 11.4 | - | - |
| Shortraker Rockfish | 14.5 ± 11.1 | 13.8 ± 10.9 | 12.9 ± 10.2 | 16.9 ± 12.3 | 14.6 ± 10.9 |
| Skate | 25.4 ± 8.3 | 28.1 ± 12.6 | 20.3 ± 11.6 | 35.3 ± 14.2 | 13.2 ± 9.3 |

*Mixing model results separating sablefish and dogfish*

Though sablefish and dogfish occupy similar isotopic space and were combined for the analysis, we ran separate mixing models here with sablefish and dogfish listed separately. Even when separated, sablefish and dogfish both had relatively high contribution to sperm whale diets, with skates being nearly as high (A4, A5). Top contributors to sperm whale diets were sablefish, rockfish, skates, and dogfish (A4).


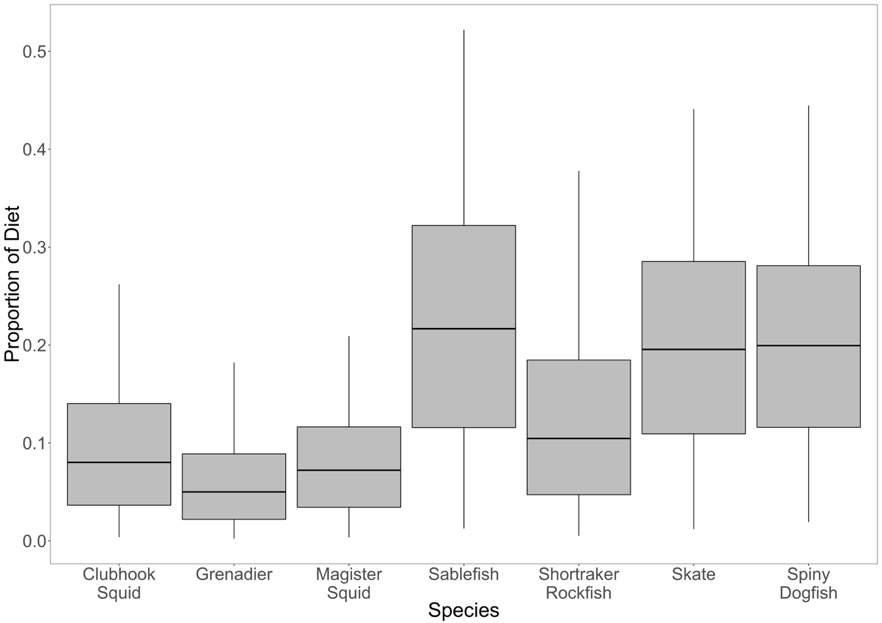


A4. Boxplots showing mixing model estimates of the proportional contribution of each prey to sperm whale diets, with sablefish and dogfish separated. Boxes represent lower and upper quartiles with a median line, while ends of whiskers show 95% credible intervals.

*Mixing model results using low and high trophic enrichment levels.*

We ran mixing models separately to explore the effect of trophic enrichment factor (TEF) on results. TEFs for δ^15^N values in the skin of free ranging pilot whales and fin whales have been estimated at 1.7‰ and 2.8‰ respectively (1,2). TEFs for δ^13^C values from each of these studies remained about 1‰. To explore how TEF estimates affected our results, we ran mixing models with each of these estimates separately. Using a lower TEF notably increased the dietary contribution of skates to sperm whale diets, and to a lesser extent clubhook squid (A5, A3). Using a higher estimate of TEF notably increased the dietary contribution of magister squid to sperm whale diets (A5, A3). Under both the low and high TEF estimates, the sablefish/dogfish group and rockfish remained higher contributors to sperm whale diet as well (A5-6, A3).


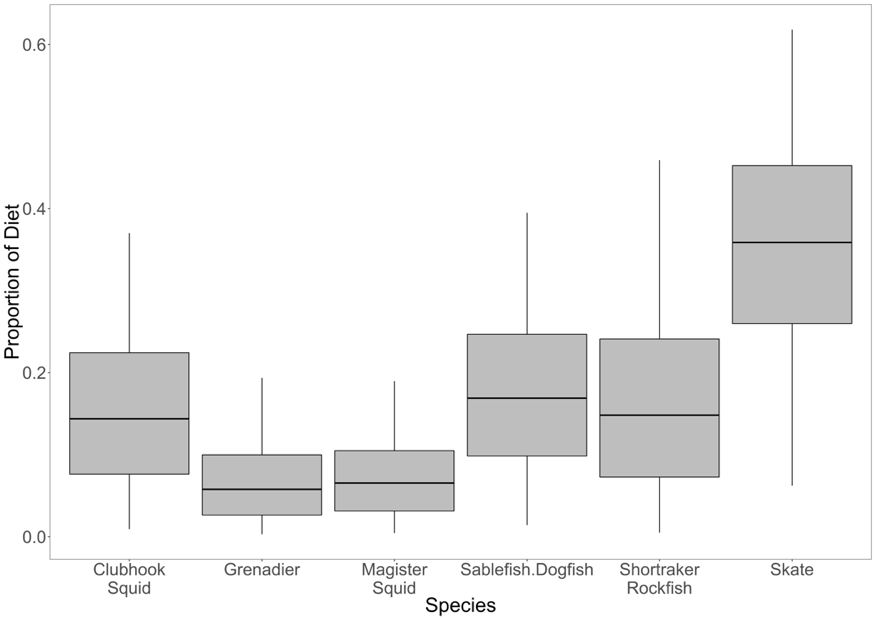


A5. Boxplots showing mixing model estimates of the proportional contribution of each prey to sperm whale diets, using a low estimate of trophic enrichment factor (TEF) of 1.7‰ for δ^15^N values. Boxes represent lower and upper quartiles with a median line, while ends of whiskers show 95% credible intervals.


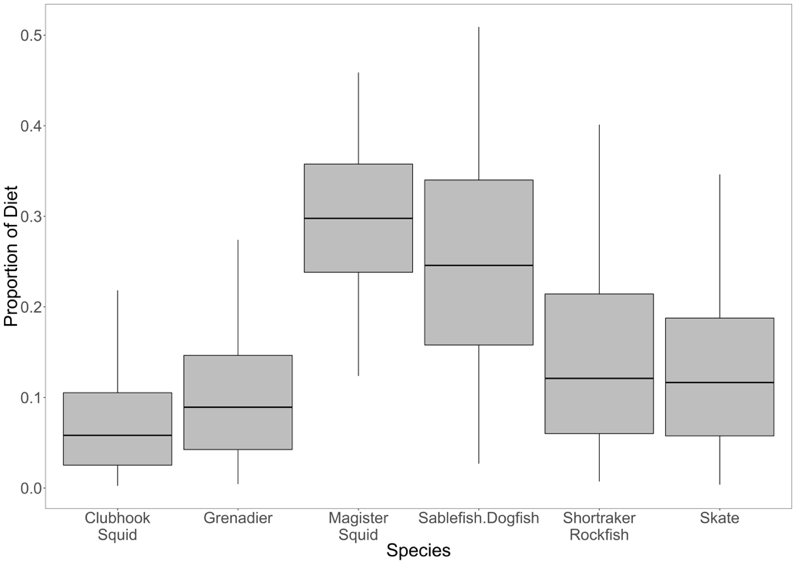


A6. Boxplots showing mixing model estimates of the proportional contribution of each prey to sperm whale diets, using a high estimate of trophic enrichment factor (TEF) of 2.8‰ for δ^15^N values. Boxes represent lower and upper quartiles with a median line, while ends of whiskers show 95% credible intervals.

Literature Cited

1. Abend AG, Smith TD. Differences in stable isotope ratios of carbon and nitrogen between long-finned pilot whales (*Globicephala melas*) and their primary prey in the western north Atlantic. ICES J Mar Sci. 1997;54:500–3.

2. Borrell A, Abad-Oliva N, Gõmez-Campos E, Giménez J, Aguilar A. Discrimination of stable isotopes in fin whale tissues and application to diet assessment in cetaceans. Rapid Commun Mass Spectrom. 2012;26(14):1596–602.
